# Detection of Antigen-Specific T Cells Using In Situ MHC Tetramer Staining

**DOI:** 10.3390/ijms20205165

**Published:** 2019-10-18

**Authors:** Hadia M. Abdelaal, Emily K. Cartwright, Pamela J. Skinner

**Affiliations:** 1 Department of Veterinary and Biomedical Sciences, University of Minnesota, St. Paul, MN 55108, USA; moham698@umn.edu (H.M.A.); cartw082@umn.edu (E.K.C.); 2 Department of Microbiology and Immunology, Zagazig University, Zagazig 44519, Egypt; 3 Microbiology Research Facility, 689 23rd Avenue SE, University of Minnesota, Twin Cities, MN 55455, USA

**Keywords:** T cells, In situ tetramer staining, MHC tetramer, immune response, antigen-specific, confocal microscopy, fresh tissue

## Abstract

The development of in situ major histocompatibility complex (MHC) tetramer (IST) staining to detect antigen (Ag)-specific T cells in tissues has radically revolutionized our knowledge of the local cellular immune response to viral and bacterial infections, cancers, and autoimmunity. IST combined with immunohistochemistry (IHC) enables determination of the location, abundance, and phenotype of T cells, as well as the characterization of Ag-specific T cells in a 3-dimensional space with respect to neighboring cells and specific tissue locations. In this review, we discuss the history of the development of IST combined with IHC. We describe various methods used for IST staining, including direct and indirect IST and IST performed on fresh, lightly fixed, frozen, and fresh then frozen tissue. We also describe current applications for IST in viral and bacterial infections, cancer, and autoimmunity. IST combined with IHC provides a valuable tool for studying and tracking the Ag-specific T cell immune response in tissues.

## 1. Introduction

T cells play a pivotal role in the adaptive immune response. They perform a wide range of immune functions, including, but not limited to, providing help for B cells, protecting against intracellular and extracellular pathogens, detecting and killing cancer cells, and preventing autoimmunity [1]. T cells recognize the antigen (Ag) via a T cell receptor-cluster of differentiation 3(TCR-CD3) complex in the context of peptide-major histocompatibility complex (p-MHC) on the surface of antigen-presenting cells (APCs). Cluster of differentiation 4 (CD4)^+^ T cells recognize antigens processed by APCs placed into a groove of MHC class II molecules (MHCII), whereas Cluster of differentiation 8 (CD8)^+^ T cells recognize antigens presented by MHC class I molecules (MHCI). Regardless of the class of MHC, TCR: p-MHC interaction is required for initiating the T cell signaling cascade, leading to T cell activation [2].

The development of flow cytometric analysis of Ag-specific CD8^+^ and CD4^+^ T cells using fluorochrome-conjugated p-MHCI and p-MHCII tetramer staining, respectively, has dramatically increased our understanding of the cellular immune response [3,4]. Using flow cytometry, we are able to determine the quantity, function, and phenotype of Ag-specific T cells [5,6,7] and identify associations between the human leukocyte antigen (HLA) haplotype and disease progression [6,7,8]. Despite these important contributions, a major limitation of flow cytometry is the inability to visualize the localization of Ag-specific T cells, both with regards to their interactions with other cells, as well as their distribution within the tissue compartment. In addition, the dissociation of tissues into a single cell suspension for flow cytometry tends to underestimate the quantity of Ag-specific T cells within non-lymphoid tissues, such as the female reproductive tract (FRT), lung, and liver [9]. Thus, while flow cytometric analysis of Ag-specific T cells is extremely valuable, it fails to determine the spatial relationships between Ag-specific T cells and target cells and underestimates their total numbers in tissue, which are crucial for a complete understanding of the cellular adaptive immune response.

We developed a method for the in situ detection of Ag-specific CD8^+^ T cells using MHCI tetramers. Using in situ MHCI tetramer (IST) staining combined with IHC, we were able to directly visualize and quantify Ag-specific CD8^+^ T cells and their specific location within the tissue compartment [10]. We used fresh, unfixed, lightly fixed, and frozen spleens from TCR transgenic mice. Ag-specific CD8^+^ T cells were readily detected in the spleens from the transgenic mice, and we found that fresh tissues by far produced the best quality staining [10]. At the same time as we were performing these studies, Haanen et al. developed and used similar IST staining methods combined with IHC to detect virus-specific CD8^+^ T cells in TCR-transgenic and wild-type virus-infected mice, as well as to detect endogenous CD8^+^ T cells directed against epitope tagged tumor cells in mice [11].

Since this time, we and others have also developed IST methods that use p-MHCII multimers to detect Ag-specific CD4^+^ cells in situ [12,13,14,15,16]. With MHC class I and class II IST technologies, we are able to determine the spatial and temporal location and abundance of Ag-specific T cell responses in tissues. By combining IST with IHC, we are able to stain Ag-specific T cells, as well as cellular markers. Additional cellular markers allow phenotypic characterization of Ag-specific T cells and the surrounding cells in the tissue, which can include target cells. We have recently produced a video demonstrating IST staining [17], and these IST staining methods have previously been reviewed [2,18,19,20]. This review article builds on previous reviews, incorporates new methodologies, and describes more recently developed applications.

## 2. In Situ Tetramer Staining

Tetramers designed for IST are the same as those used in conventional flow cytometry. They both consist of four MHC monomers loaded with a specific peptide to interact with the T cell specific to that peptide [3].

## 3. Direct vs. Indirect IST

The two common methods used to detect Ag-specific T cells in situ are direct and indirect IST. Direct IST requires the use of MHCI or MHCII tetramer conjugated directly to a bright fluorophore, like allophycocyanin (APC) or phycoerythrin (PE) to directly label Ag-specific T cells [11,21,22]. Direct staining can also be done by using MHC-dextran multimers (dextramers) [16,23]. These multimers have more p-MHC complexes and more fluorochromes, allowing for brighter signal than standard tetramers with only one fluorophore. In addition, Tjernlund et al. used Qdot 655 multimers to directly detected SIV-specific CD8^+^ T cells [7,24].

**Figure 1:**
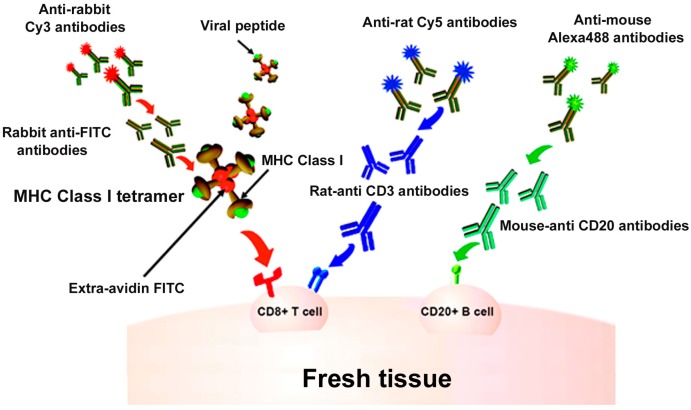
In situ major histocompatibility complex (MHC) class I (MHCI) tetramer staining combined with immunohistochemistry (IHC) to detect virus-specific CD8^+^ T cells. Schematic diagram of in situ MHC tetramer (IST) combined with IHC to detect virus-specific CD8^+^ T cells in fresh, unfixed tissue sections. An MHCI tetramer consists of four biotinylated MHC-class I monomers loaded with a viral peptide (or another antigenic peptide) bound to a fluorescently labeled avidin molecule. After primary incubation with MHCI tetramers, sections are fixed and then anti-FITC antibodies are used to amplify the tetramer signal. This signal is then further amplified using Cy3-tagged anti-Rabbit IgG antibodies. Sections can be counterstained with CD3 antibodies to label T cells (blue), and CD20 antibodies to label B cells (green).

**Figure 2:**
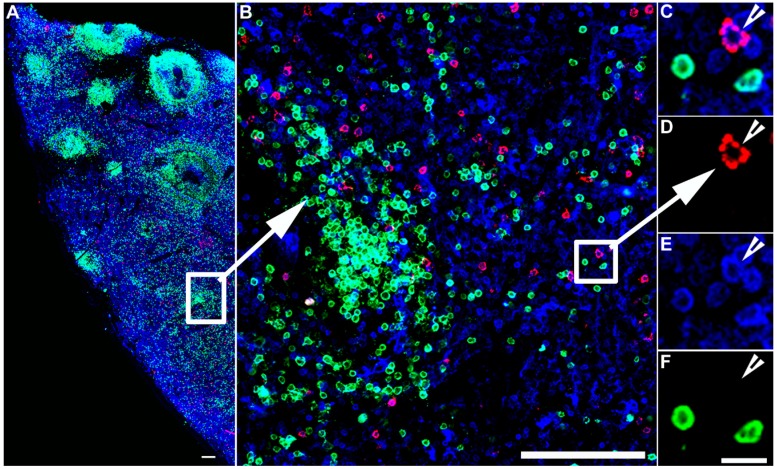
IST detection of virus-specific CD8^+^ T cells. IST Combined with IHC in spleen sections from an SIV infected rhesus macaque. Fresh unfixed spleen section was stained with Mamu-A*01 tetramers loaded with SIV Gag/CM9 peptides detect SIV-specific CD8^+^ T cells (Red color), and counterstained with CD3 antibodies to label T cells blue, and CD20 antibodies to label B cells green and delineate B cell follicles. Confocal images were collected using a 20 X objective and 3 μm z-steps. (**A**) shows a montage of several projected confocal z-series fields. The scale bar = 100 μm. (**B**) shows an enlargement of the selected area in panel (**A**), which is a confocal Z-scan showing the distribution of tetramer^+^ T cells within the spleen. The scale bar = 100 μm. (**C**–**F**) are enlargements for the selected area in panel B and shows that an SIV-specific CD8^+^ T cell is tetramer^+^ (**C**,**D**), CD3^+^ (**E**), and CD20^−^ (**F**), scale bars = 10 μm. Arrowheads point to a virus-specific CD8^+^ T cell.

Indirect IST uses antibody staining directed against the fluorophore on the tetramer to amplify the signal [10,14,17,25,26,27,28,29,30,31,32,33,34,35,36]. For example, as described in Figure 1, the four biotinylated monomers are bound to an FITC-labeled ExtrAvidin molecule (a fluorescently labeled avidin). Conjugation to this FITC-avidin molecule allows amplification of the tetramer signal using an anti-FITC antibody. In this case, tissue is labeled with FITC-conjugated MHCI tetramers, followed by incubation with rabbit- α-FITC antibodies for signal amplification. Then, a secondary antibody, such as Cy3 labeled α-rabbit IgG, further amplifies the signal. Figure 1 also shows concurrent staining with CD3 antibodies to label T cells (in blue) and CD20 antibodies to label B cells (in green). Figure 2 shows a representative image of a spleen tissue section stained indirectly with FITC-conjugated MHCI tetramers to detect virus-specific CD8^+^ T cells (in red) and counterstained with antibodies against CD3 to label T cells (in blue) and CD20 to detect B cells (in green). 

MHC tetramers conjugated to PE and APC can similarly be used for indirect staining [21,22,37,38,39,40,41]. In addition, antibodies directed against streptavidin can be used. For example, Vries et al. used indirect MHCI IST to detect melonoma-specific CD8^+^ T cell following dendritic cell vaccination of melanoma patient, where they used a rabbit anti-streptavidin that recognizes MHCI tetramer-associated streptavidin molecules. They amplified the signal from the anti-streptavidin antibodies using goat-anti-rabbit Alexa594 [42]. Another application of indirect tetramer staining involves the use of the horseradish peroxidase (HRP)-conjugated tetramer. Instead of a fluorochrome, Yang et al. used tetramers conjugated to HRP–streptavidin and amplified the signal with the addition of biotin-conjugated tyramide [21,43].

Both methods have their advantages and drawbacks. Direct staining is a simpler procedure, can result in lower background staining, and provides more options to co-label other proteins since no secondary antibody is involved in labeling TCRs. However, direct staining provides a weaker signal intensity and is relatively more expensive because it requires as much as 40 times the tetramer of the indirect staining method [18]. In contrast, indirect labeling is a multi-step procedure that is more time consuming. Indirect staining, however, yields a more intense signal, resulting in a much higher signal to noise ratio and is relatively less expensive because it requires smaller amounts of the tetramer reagents.

## 4. IST Staining on Fresh and Frozen Tissue

IST staining can be done on fresh tissue sections, fresh then frozen tissue, or frozen tissue sections. In situ tetramer staining is ideally performed using unfixed, fresh tissue sections to maintain the structure and mobility of TCRs to interact with p-MHC tetramers [10,11]. To generate fresh 200 μm tissue sections, either a Vibratome or Compresstome can be used. However, a Compresstome is much more efficient in generating sections and accommodates larger section sizes [25]. While fresh tissue sections are ideal, there are some circumstances where fresh samples are not feasible. For example, some studies require that samples be shipped overnight. Some studies have limited tissue sampling, size availability, or their tissue was already frozen and archived. To determine if these conditions were feasible to perform IST, we performed IST on tissue samples that were stored at 4 °C overnight in PBS, lightly pre-fixed or frozen [10]. We found that there was no difference in the quality of the staining that was done on either spleen sections directly after dissection or spleen sections that were stored overnight in PBS at 4 °C. Moreover, we found that the IST also worked on lightly fixed spleen tissue from TCR transgenic mice (defined as 2% formaldehyde or 50% methanol and 50% acetone). While the IST worked on lightly fixed tissues, it yielded a higher background and less intense signal than the fresh, unfixed tissue. Additionally, IST worked on 10 μm-thick frozen sections but also resulted in weaker signal intensity compared to that from fresh tissue section [10]. Vyth-Dreese et al. compared direct tetramer staining on fresh viable tissue sections versus cryopreserved tissue sections and was able to detect Ag-specific T cells only in viable tissue sections. However, they were able to detect Ag-specific T cells in fresh skin tissue sections that were pre-stained with tetramers and then cryopreserved [22]. Similarly, others have now successfully performed indirect IST on fresh tissue pre-incubated with tetramers, and then fixed and snap-frozen. Later, frozen sectioning was done followed by IHC and tetramer amplification [38,44].

For staining frozen sections, IST has been described on unfixed and fixed tissue samples. Fixation was done before tetramer incubation, after tetramer incubation, or fixed both before and after tetramer incubation. We performed IST on unfixed spleen tissue stored in OCT freezing medium, and fixation was done post-tetramer incubation [10]. Similarly, Oerke et al. and Tully et al. used indirect IST using PE tetramers on frozen sections, and fixation was done after tetramer incubation [40,41]. Tjernlund et al. used Qdot 655 multimers to directly stain frozen section where fixation was done post-tetramer incubation [7,24]. In addition, Yuhong et al. used indirect IST to detect *Mycobacterium tuberculosis* (*M. tb*)-specific CD4^+^ T cells in lymph node and lung from untreated *M. tb* patients. For this study, IST was done on frozen sections that were first fixed in 4% PFA then incubated overnight with tetramer [39]. Similarly, Vries et al. lightly fixed frozen tissue sections before starting IST and fixed them again after incubation with tetramers [42].

In summary, performing IST on fresh (not frozen) unfixed tissue has several advantages compared to pre-fixed or thin frozen sections. IST with fresh tissue sections results in the highest staining intensity over background fluorescence. In addition, the use of fresh 200 μm-thick sections provide more information about the tissue because it allows the examination of 20 times more tissue than a thin 10 μm-thick frozen sections. When trying to detect a rare population of Ag-specific T cells, the more tissue examined the greater chances are of detecting rare cells. Moreover, the thick fresh sections can be examined using confocal microscopy to provide a 3-D view of the location and the interaction of Ag-specific CD8^+^ T cells with other cells and tissue structures. On the other hand, frozen sections offer some great advantages in that they enable the detection of Ag-specific T cells in archived samples. Additionally, with frozen sections, tissue samples can be stored and processed when needed, which makes it easier to answer future questions that might arise.

## 5. Specificity and Sensitivity of IST

A critical and remarkable property of the cellular adaptive immune response is specificity, where selective activation and expansion of a very small population of Ag-specific T cells is required. Therefore, the specificity and sensitivity of IST is the key to its success in detecting such small fractions of Ag-specific T cells [4]. Because high background autofluorescence is inherent when imaging whole tissues, ensuring the proper negative controls for IST staining is crucial [10]. There are several methods used to confirm the specificity of tetramer staining using IST. In the interaction between the p-MHC complex and TCR, both the amino acid sequence of the peptide and the haplotype of MHC are critical for determining specificity of the T cell. Both of these variables can be altered in an experiment to ensure the tetramer staining is specific. When changing the amino acid sequence of the peptide, the same fluorescently labeled MHC molecule is used, but the peptide loaded should not be present in the experimental system. For example, in studies of Mamu-A*001:01 rhesus macaques, FITC-labeled Mamu-A*001:01 tetramers loaded with an irrelevant peptide FV10 (FLPSDYFPSV), a peptide from the hepatitis B viral core protein served as a negative control for FITC-labeled MHCI Mamu-A*001:01 SIV GagCM9 (181–189) (CTPYDINQM) tetramers and for FITC-labeled MHCI Mamu-A*001:01 SIV Tat STPESANL (SL8) tetramers [17,29,30,35]. As another example, a study using tissues from human study participants used HLA- B*57 tetramers loaded with MART (ELAGIGILTV), a peptide from melanoma protein, to serve as a negative control for FITC-labeled HLA- B*57 HIV-1 Gag IW9 (ISPRTLNAW) or QW9 (QASQEVKNW) tetramers during detection of HIV-specific tissue-resident CD8^+^ T cells within the gastrointestinal tract in a chronic infection [27].

Alternatively, studies have revealed specificity by using MHC-mismatched tetramers. These are tetramers loaded with the peptide of interest but not able to bind to T cells in the tissue due to cells in the tissue expressing different MHCI molecules [10,11]. A third type of negative control includes using a tissue that does not have Ag-specific cells of interest. In this case, the tissue and tetramer are the same haplotype, but the individual animal or study participant that was sampled was not infected with microbes [10,18,21].

We found that the specificity and sensitivity of IST staining is comparable to that of flow cytometry. Following the adoptive transfer of transgenic T cells into a wild-type mouse, the spleen of the recipient mouse was split in half. One half was used to determine the number of tetramer^+^ CD8^+^ T cells using flow cytometry, and the other half was used for IST staining. Both techniques showed that ~1% of the CD8^+^ cells were tetramer^+^ [10]. Haanen et al. found that the background staining in tissues permits detection limits of 0.1–1% of T cells, whereas the limit of detection of flow cytometry is less than 0.1% [13]. Nonetheless, the sensitivity of IST is sufficient to detect endogenous antigen-specific T cell responses.

## 6. Applications for In Situ Tetramer Staining

As mentioned previously, traditional tissue processing for MHC tetramer staining by flow cytometry requires dissociation of the tissue into a single cell suspension. Though flow cytometry is powerful in providing information about the phenotypes of Ag-specific cells, it does not show where specifically in the tissue they are located, what the phenotype of cells are in specific locations or what cells they are interacting within specific tissue compartments. This information can be critical for understanding T cell responses to infections, predicting vaccine efficacy, and investigating an immune response within the tumor microenvironment.

In HIV and SIV infection, CD4^+^ T follicular helper (T_FH_) cells are a major site of viral persistence during antiretroviral therapy and are a critical barrier to eradication [34,45,46,47,48,49,50]. Studies using IST have shown that low levels of virus-specific CD8^+^ T cells in lymphoid follicles permit ongoing viral replication in T_FH_ [33,36]. This knowledge can inform future vaccine and cell therapy design for HIV infection by monitoring or inducing the accumulation of HIV-specific CD8^+^ T cells in B cell follicles [17,28,29,34,35,36,51].

Another application combines IST with in situ hybridization (ISTH) to determine a direct spatial and temporal relationship between virus-specific CD8^+^ T cells (effector cells) and virus-infected target cells. Looking at both the SIV infection of non-human primates (NHP) and LCMV infection in mice, it was determined that the location, timing, and abundance of antigen-specific T cells directly relates to the number of infected target cells [26,52].

In barrier tissues, like the lung, gut, and skin, CD4^+^ and CD8^+^ T cells take up residence following an infection. These tissue resident T cells (T_RM_), are the first line of defense against secondary pathogen exposure. IST staining has been used to increase our understanding of the immune response at these sites, including cell types required for the generation and maintenance of T_RM_. In a murine model of HSV-2 infection, IST staining was used to determine CD301b^+^ dendritic cells (DCs) are critical for initiating and maintaining the CD8^+^ T_RM_ population in the female reproductive tract (FRT) [53]. They show that activation by CD301b^+^ DCs activates CD8^+^ T_RM_ to produce interferon-gamma (IFN-γ), and this response is necessary for protection from HSV’s challenge.

IST staining has also been used to visualize in situ dynamics of the immune response to *Listeria monocyotogenes* (LM) [54]. As early as day 3 post-infection, LM-specific CD8^+^ T cells are detected in the spleen of infected mice and located at the border of the T and B cell zones. They also show an interaction of LM-specific CD8^+^ T cells with CD11c^+^ DCs in clustered foci within the T cell zones. Interestingly, after both influenza and LM infection, memory Ag-specific CD8^+^ T cells can be found within B cell follicles. Upon a secondary challenge, there are many more foci of Ag-specific CD8^+^ T cells within T cell zones, consistent with a robust CD8^+^ memory T cell response to the secondary challenge.

Outside of infectious diseases, IST staining can be applied to other fields, including cancer biology and autoimmunity. In one study, IST was used to determine the feasibility of a dendritic cell-based vaccine for melanoma. In the three study participants examined, researchers detected tumor-specific CD8^+^ T cells within the tumor following vaccination [42]. This has important implications for predicting the efficacy of cancer vaccines, as circulating T cell responses are not always a good indicator of protection. In another study, researchers investigated the CD8^+^ T cell response in type 1 diabetes (T1D) [55]. Using IST, they described the first confirmation of Ag-specific, autoreactive CD8^+^ T cells in the islet lesions from T1D patients. Interestingly, they also showed that recent onset patients (<1 year duration of the disease) had a more clonally restricted CD8^+^ T cell response in the islets, whereas long standing patients (>1 year duration of the disease) had a more diverse CD8^+^ T cell response [55]. Much like predicting vaccine efficacy, understanding the immune response at the site of autoimmunity, and not just in peripheral blood, is critical to improving future treatment of cancer and autoimmune diseases.

While the bulk of IST staining has been done using MHCI tetramers to study the CD8^+^ T cell response, MHCII tetramers are available to investigate the CD4^+^ T cell response to infections and autoimmunity. In one study examining patients with active *Mycobacteriaum tuberculosis* (*M. tb*) infection, IST staining showed Ag-specific CD4^+^ T cells producing IFN-γ and TNF-α in the lymph nodes, lung granulomas, and cavernous tissue [12]. In experimental autoimmune encephalomyelitis (EAE), antigen-specific CD4^+^ T cells were detected in the lymph nodes and central nervous system (CNS) of diseased animals, but not in uninfected animals [13].

## 7. Limitations of IST Staining

While there are many important applications for IST staining, this technique has significant limitations. Many of these limitations are a factor of tetramer technology. Unlike a peptide pool, used to broadly probe an antigen-specific T cell response, a tetramer can only have one peptide presented and, therefore, will only interact with T cells specific for that peptide. This can potentially cause researchers to underestimate the total immune response to a pathogen or vaccine, and similarly, can lead to over interpretation of results for that epitope. Most often, tetramers are made against the immunodominant epitope. The immunodominant epitope is the peptide that the majority of the CD4^+^ or CD8^+^ T cell response is generated against. However, there are often subdominant responses that might be overlooked with tetramer staining.

Because tetramer technology takes advantage of the specific interaction between the p-MHC complex and the TCR, use of the technology requires determining the MHC genotype of an individual prior to examining the Ag-specific response. There may be limitations in the availability of tetramers for MHC molecules encoded by a particular allele. Additionally, there is the possibility that the immune response you are visualizing cannot be generalized to other MHC molecules. For example, people expressing *HLA-B27* and *-B57* MHC molecules are more often elite controllers of HIV infection [56,57]. In rhesus macaques, Mamu-A*001:01 [58], -B*008:01 molecules [59], and -B*017:01 [60] alleles are also associated with enhanced control of SIV infection. While understanding the immune response in elite controllers of HIV/SIV infection is valuable information, it is not generalizable to all CD8^+^ T cell responses during HIV/SIV infection. 

Another limitation that can be a problem for the in situ visualization of Ag-specific CD4^+^ T cells is the affinity threshold. The affinity threshold required for staining of the p-MHC with tetramer is higher than that required for activation of TCR, which biases tetramer technology to detect primarily high affinity T cells [61]. Work in recent years has shed light on low affinity T cells contribute significantly to the immune response [62,63]. Though this has been described primarily for Ag-specific CD4^+^ T cells, it can be found in Ag-specific CD8^+^ T cells, as well [64]. Researchers have begun to address this limitation by increasing the number of p-MHC complexed in multimers. They have changed the scaffold from biotin to dextran which allows more p-MHC and more fluorescent molecules to bind, both of which help increase the detection of antigen-specific T cells [65].

One of the challenges unique to IST staining is that the best results are obtained using fresh tissue samples [18,21]. We showed that while fixed and frozen tissue can be used in IST, the best results are gathered from fresh, unfixed samples [10]. Nonetheless, sometimes experimental conditions do not allow for the use of fresh, unfixed samples.

We have not been successful at getting IST staining to work well and consistently in different experimental systems with fixed or frozen tissues. However, as mentioned above, others have demonstrated success in their experimental systems. Certainly, having a robust, reliable method to track and phenotype Ag-specific T cells in fixed and frozen tissues would be a great advantage to the scientific community. Advancements in existing IST staining methodologies, or the development of new methods, are warranted to achieve this goal. Future methods to detect Ag-specific T cells in fixed and frozen tissues may be on the horizon and may not rely on MHC-tetramers or multimers. For example, in situ hybridization methods that detect the unique hypervariable regions of TCR genes, termed complementarity-determining regions (CDRs), might be an effective means to track Ag-specific T cells in fixed and frozen sections. Indeed, Advanced Cell Diagnostics have recently developed an in situ hybridization method called BaseScope that may allow detection of CDRs.

In summary, IST combined with IHC has radically enhanced our understanding of the Ag-specific T cell response. Not only does it enable the determination of the magnitude and phenotype of Ag-specific CD4^+^ and CD8^+^ T-cell responses in situ, but it also is a critical tool in tracking their location within tissue compartments and cell–cell interactions. IST staining has been, and continues to be, used to enhance our understanding of the local cellular immune response in many areas of research, including cancer biology, vaccinology, viral pathogenesis, bacterial infection, and autoimmune diseases.
